# Fabrication of Cu-Al-Mn-Ti Shape Memory Alloys via Selective Laser Melting and Its Nano-Precipitation Strengthening

**DOI:** 10.3390/mi16080857

**Published:** 2025-07-25

**Authors:** Lijun He, Yan Li, Qing Su, Xiya Zhao, Zhenyu Jiang

**Affiliations:** 1 Hubei Engineering Research Center for BDS-Cloud High-Precision Deformation Monitoring, Artificial Intelligence School, Wuchang University of Technology, Wuhan 430223, China; allround@163.com; 2 Institute for Advanced Marine Research, China University of Geosciences, Guangzhou 511462, China; 3 Gemmological Institute, China University of Geosciences, Wuhan 430074, China; 13138596260@163.com (Q.S.); 20161002733@cug.edu.cn (X.Z.); jiangzhenyu@cug.edu.cn (Z.J.)

**Keywords:** selective laser melting, Cu-based shape memory alloys, microstructures, nano-precipitation strengthening

## Abstract

A Cu-11.85Al-3.2Mn-0.1Ti shape memory alloy (SMA) with excellent superelasticity and shape memory effect was successfully fabricated via selective laser melting (SLM). Increasing the energy density enhanced grain refinement, achieving a 90% refinement rate compared to cast alloy, with an average width of ~0.15 µm. Refined martensite lowered transformation temperatures and increased thermal hysteresis. Nanoscale Cu_2_TiAl phases precipitated densely within the matrix, forming a dual strengthening network combining precipitation hardening and dislocation hardening. This mechanism yielded a room-temperature tensile strength of 829.07 MPa, with 6.38% fracture strain. At 200 °C, strength increased to 883.68 MPa, with 12.26% strain. The maximum tensile strength represents a nearly 30% improvement on existing laser-melted quaternary Cu-based SMAs.

## 1. Introduction

Shape memory alloys (SMAs) are smart materials with the function of recovering the inelastic strains induced by external forces and returning them to their original forms. Due to their notable attributes—specifically, shape memory effect (SME) and super elasticity (SE) [1,2,3,4]—SMAs have found extensive applications across diverse fields, including aerospace [5], automotive [6], biotechnology [7], robotics [8], solid-state refrigeration [9,10], and smart wearable devices [11,12]. Copper-based shape memory alloys (Cu-based SMAs), as the first member of the SMA family [13], exhibit great SME and SE properties, with low cost and easy processability. A series of Cu-based SMAs have been developed in the past few decades, including Cu–Al–Mn [14,15,16,17], Cu–Al–Ni [18,19,20], and Cu–Zn–Al alloys [21,22,23]. However, the inevitable brittleness of Cu-based SMAs induced by the highly ordered structure of the parent phase hinders their further applications. Several manufacturing technologies have been applied in an attempt to improve the mechanical performance of Cu-based SMAs by restraining the formation of grain boundary triple junctions, for instance, the Taylor–Ulitovsky method [24,25,26], the unidirectional solidification technique [27,28], and the silica-gel bead infiltration method [29,30]. Nevertheless, some drawbacks, like the non-customizable geometrics and time-consuming nature of the process, are noticeable during the above-mentioned fabrication techniques. Therefore, a novel and efficient manufacturing method is urgently required to fabricate Cu–based SMAs.

Selective laser melting (SLM) is recognized as the most commonly used additive manufacturing (AM) technology in metalworking procedures, due to its exceptional laser quality and high fabricating precision. This technique stands out for its proficiency in achieving the integration of materials, structures, and performances [31], commonly denoted as three-dimensional printing (3-DP) technology. In our previous works, SLM has been successfully utilized for the manufacturing of metal matrix Ag-Cu/diamond composites [32], as well as high-reflectivity and thermal conductivity Ag-Cu multi-material structures [33]. Some researchers have also proven that it is feasible to fabricate Cu-based SMAs via SLM in recent years. Gustmann et al. [34,35] obtained fully martensitic samples with a high density of up to 99% via parameter optimization and remelting steps in the SLM process of Cu-Al-Ni-Mn SMAs. However, the tension properties were less ideal and did not drastically change after the remelting procedure. Tian et al. [36] fabricated Cu-Al-Ni-Ti SMAs via SLM and pointed out that the grain refinement as well as the suppression of the brittle γ_2_ phase resulted in better tensile properties than with the cast ones. Zhuo et al. [37] investigated the effect of the element evaporation of SLMed Cu-Zn-Al SMAs and found that the Zn content decreased with the increase in energy density, and thus the predominated phase constitution changed from the needle-like β’ martensite phase to the rod-like or even equiaxed α phase, resulting in a lower microhardness and a higher irrecoverable strain, which was harmful for the alloys. Babacan et al. [14] fabricated Cu-Al-Mn SMAs with a strong [001] texture perpendicular to the building direction and significantly enlarged the recoverable strain by applying scan vector rotations of 90° during the SLM process. These works illustrate that it is possible to fabricate Cu-based SMAs with high densities and controllable structures. In addition, some efforts have been implemented to increase the shape memory effect of Cu-Al-Ni SMAs through the partial reinforcement of accumulating localized residuals by adding particles like Al_2_O_3_ [38] and graphene [39]. Furthermore, four-dimensional printing (4-DP), as a pre-programmed process that can respond to external stimuli over time, has been applied in Cu-based SMAs to improve mechanical properties as well. For example, the 4-DP of two separate structures with different transformation temperatures was used to enhance the shape recovery of Cu-Al-Ni SMAs [40], and corrugated structural Cu-Al-Mn-Ti SMA components fabricated via 4-DP achieved a shape memory recovery rate of up to 100% at a pre-strain of 6% [41]. However, Zhang et al. [20] revealed that Cu-Al-Ni SMAs fabricated via the SLM process have an intrinsic characteristic under tensile-compressive loading, tension–compression asymmetry, which means excellent compressive but poor tensile properties. Nevertheless, in most of the application areas, tensile properties are much more important than their counterparts, compressive properties. Therefore, it is essential to improve the tensile properties of Cu-based SMAs.

Here, the Cu-11.85Al-3.2Mn-0.1Ti (wt.%) shape memory alloy with enhanced tensile properties was fabricated via SLM technology. The process parameters, microstructure characteristics, phase transformation, and mechanical properties were investigated. Moreover, the underlying mechanism for the enhanced tensile properties was revealed as well.

## 2. Materials and Methods

### 2.1. Materials

The alloy powders used for the SLM process were manufactured from the Cu-11.85Al-3.2Mn-0.1Ti ingots using Ar gas atomization technology by Shandong Lianhong Tech. Co., Ltd. in Zaozhuang, China. The nominal chemical compositions of the original powders are listed in Table 1. A prior procedure should be exerted, i.e., the alloy powders should be dried in the oven at 80 °C for 10 h and then passed through a sieve (300 mesh) before the SLM process to eliminate the surface moisture and improve the fluidity of the alloy powders. The discrepancy of Ti arises from the inherent fluctuation during the powder atomization process. Although the target composition was designed as 0.1 wt.% Ti (micro-alloyed), the actual value was 0.17 wt.%. This slight increase is within the acceptable deviation range for commercial powder fabrication and does not alter the intended micro-alloying effect or phase formation behavior.

### 2.2. SLM Process

**Figure 1:**
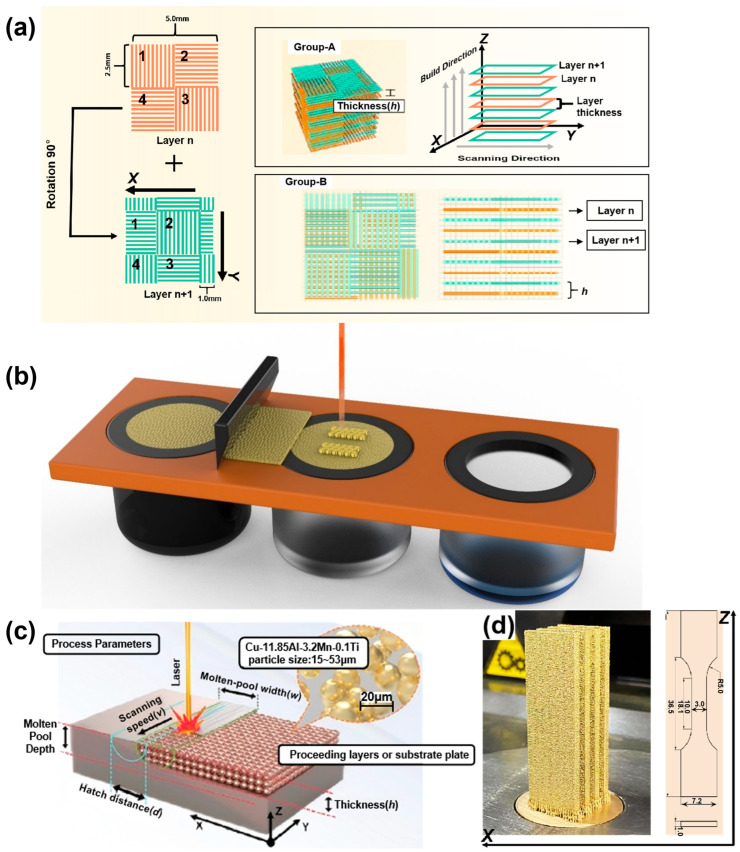
Schematic of SLM process. (**a**) Checkerboard scanning strategy; (**b**) Illustration of laser melting process; (**c**) SLM-ed Cu-Al-Mn-Ti samples; and (**d**) The dimensions of tensile test samples.

All samples were fabricated in a high-purity argon atmosphere via a SISMA MYSINT100 system (SISMA, Vicenza, Italy) equipped with an Nd: YAG fiber laser (Figure 1). The parameters were listed as below, with a wavelength of 1.06 μm, maximum laser powder of 200 W, spot diameter of 30 μm, and scanning spacing of 0.1 mm. The checkerboard strategy was applied in this research: each layer was divided into square scan units of 1 mm × 1 mm, and between two adjacent layers, the scanning pattern was rotated by 90°. The scanning origin was offset by 1.0 mm in both the X and Y directions relative to the previous layer. This strategy is commonly adopted to reduce residual stress and improve metallurgical bonding between adjacent melt pools. Bulk samples with dimensions of 5 mm × 5 mm × 5 mm were fabricated for the characterization of density, microstructure, and hardness. Slab samples with a dimension of 52 mm × 17 mm × 2.4 mm were built for the mechanical tests. The SLM processing parameters of the samples are listed in Table 2.

### 2.3. Characterization Techniques

Scanning electron microscopy (SEM, FEI QUANTA 200, FEG, Hillsboro, OR, USA) with energy-dispersive X-ray spectroscopy (EDS, EDAX GENESIS 60S, Pleasanton, CA, USA) was utilized on the powders to examine the morphology and elemental distribution of the powders.

The particle size distribution was measured using a laser diffraction particle size analyzer (BT-9300HT, Bettersize, Dandong, China). An analytical balance scale (BSA223S, Sartorius, Gottingen, Germany) was used to measure the density of bulk samples based on the Archimedes’ principle. These bulk samples were mechanically polished to 2000 mesh and then polished using 0.5 μm diamond suspensions. After that, some of them were etched using a 5 g FeCl_3_·6 H_2_O + 10 mL HCl + 100 mL H_2_O mixed solution for 14s for the observation of martensite.

An optical microscope (OM, Nikon ECLIPSE Ts2, Tokyo, Japan) was utilized to observe the defects of the bulk samples. Scanning electron microscopy (SEM, ThermoFisher Apreo S, Waltham, MA, USA) was applied for the identification of martensite morphology.

A transmission electron microscope (TEM, Thermo Fisher Scientific Talos™ F200X, Waltham, MA, USA) was utilized to investigate the nano-microstructure and element distribution. The sample slice for TEM observation was milled to a thickness of 41–58 nm using a focused ion beam (FIB, Helios 5 UX, Orleans, MA, USA).

The phase composition of the samples was identified via X-ray diffraction equipment (XRD, Bruker D8 Advance, Karlsruhe, Germany) with Cu–Kα_1_ radiation. The phase transformation temperatures were determined via differential scanning calorimetry (DSC, TA DSC25, New Castle, DE, USA) at a heating and cooling rate of 10 °C/min, with a wide temperature range from 30 to 500 °C.

A hardness tester (VH1102, Wilson, Norwood, MA, USA) was utilized to measure the microhardness of the samples at a force of 0.1 kg for 10 s, and the results were reported in Vickers Hardness (VH) by the machine.

Tensile testing according to the ASTM standard was performed by a mechanical testing machine (TSE504C WANCE, Shenzhen, China) equipped with an oven at room temperature (RT) and 200 °C (HT). The test was adopted at a tensile rate of 0.5 mm/min and the strain was recorded via a laser extensometer. The tensile samples were cut from the slab samples according to the dimension (Figure 1d). Three samples were measured each time and the average results were reported. After that, the fracture morphologies were observed via scanning electron microscopy (SEM, FEI QUANTA 200, FEG, Hillsboro, OR, USA).

## 3. Results and Discussion

### 3.1. Powder Analysis and Optimization of Process Parameters

**Figure 2:**
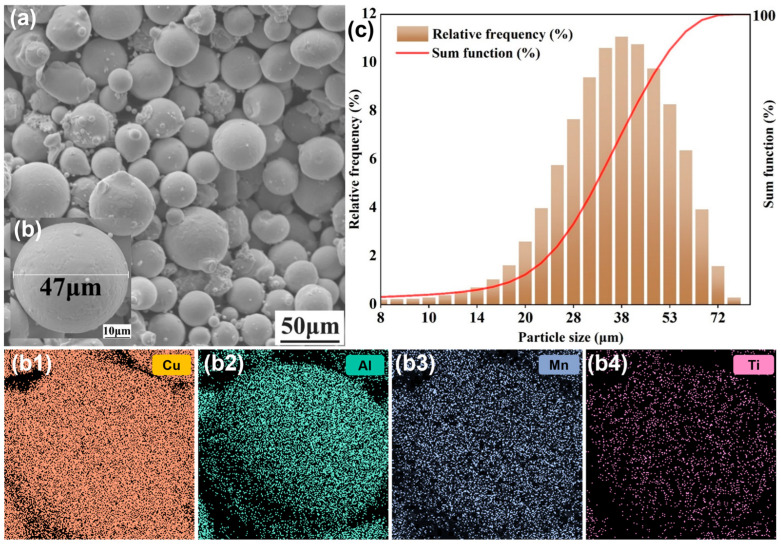
Microscopic properties of Cu-11.85Al-3.2Mn-0.1Ti powder: (**a**) SEM image of the powder morphology; (**b**) element distribution of Cu (**b1**), Al (**b2**), Mn (**b3**), and Ti (**b4**); and (**c**) particle size distribution in volume.

The quality of the powder and the optimization of parameters play a crucial role in the SLM process because defects like pores and cracks are inevitable, resulting from the high energy density and small laser spot properties of SLM [42]. Figure 2a shows the morphology of the SLM powder. Clearly, most of the powders had a spherical shape, a few of them displayed an irregular state, and a minority of them were tiny spherical powders filling in the spaces. A homogeneous elemental distribution of Cu, Al, Mn, and Ti was observed in the powder (Figure 2b), which were represented by different colors. The diameter of the powder ranged from 8 to 72 μm and clustered at around 36.85 μm (D_50_), as displayed in Figure 2c. The prior procedure ensured the preferable flowability of the powder.

Based on the morphology and width of the single track (please refer to Figure S1 in the Supplementary Information), various parameters were chosen to fabricate the bulk samples, as listed in Table 2. The volumetric energy input ($E_{v}$) was estimated as follows:

(1) $$E_{v}\;=\;\frac{P}{v\;\times \;h\;\times \;l}$$

where *P* represented the laser power, *v* represented the scanning speed, *l* represented the layer thickness, and *h* represented the hatch space.

**Figure 3:**
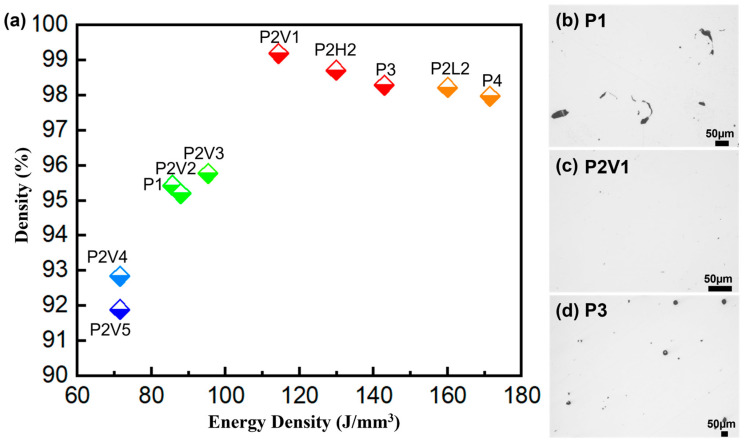
(**a**) Influence of the volumetric energy input on the average relative density of SLM bulk samples; corresponding morphology of pores of parameters (**b**) P1, (**c**) P2V1, and (**d**) P3.

The average relative densities were tested and calculated as listed in Table 2. The volumetric energy input (Equation (1)) correlated with the average relative density of the bulk parts is indicated in Figure 3a. The average relative density of the samples experienced a rise with the increasing energy input, and reached a maximum average relative density of 99.19% at the energy input of 114.29 J/mm^3^. The built parameters were *P* = 80 W, *v* = 200 mm/s, *h* = 0.1 mm, and *l* = 0.035 mm, and then a declining trend was found with the steady increase in the energy input. This trend was also illustrated by the morphologies of the samples built with energy inputs of 85.71 J/mm^3^ (Figure 3b), 114.29 J/mm^3^ (Figure 3c), and 142.86 J/mm^3^ (Figure 3d). When the energy input was quite low, irregular pores could be found in the weak-melted sample P1 due to the lack of energy, resulting in incomplete melted defects. With the rise in energy input, only micro-spherical pores were observed in the well-melted sample P2V1, leading to a minimum porosity and a maximum density. A huge number of large spherical pores started to appear in the over-melted sample P3, which indicated that the molten pool had changed from the conduction mode molten pool to the “keyhole” mode one [43], causing the decrease in the relative density in the sample. The study on the optimization of the SLM process parameters provides technical reference for fabricating Cu-based SMAs via selective laser melting technology, and an appropriate volumetric energy input is necessary to build high-density ones.

### 3.2. Microstructural Analysis

**Figure 4:**
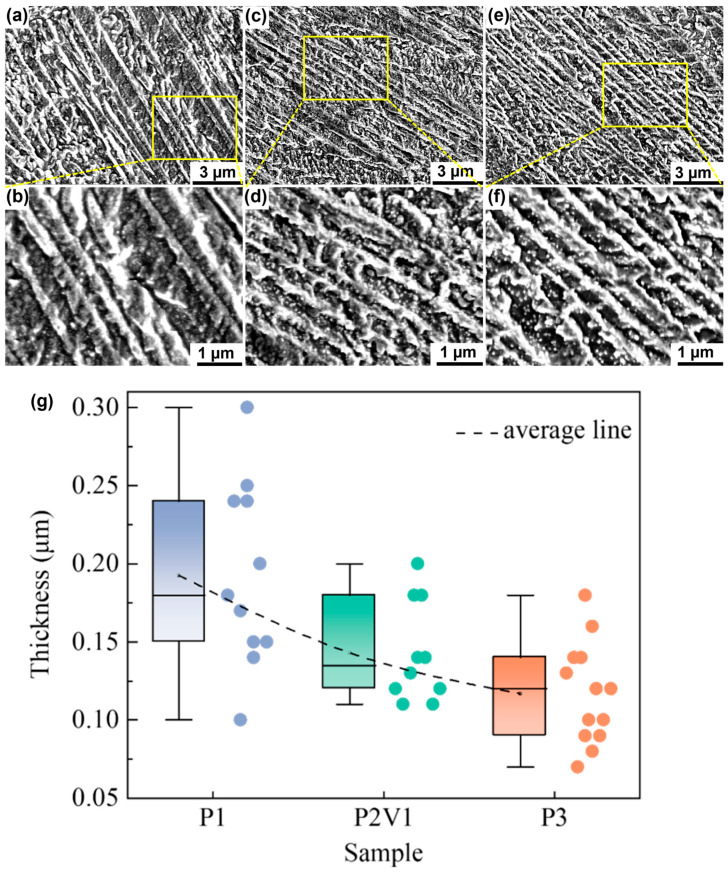
SEM images of martensite microstructures obtained with the parameters of (**a**) P1, (**c**) P2V1, and (**e**) P3, and their detailed views (**b**,**d**,**f**); (**g**) statistical diagram of the martensite widths counted from the detailed views.

Figure 4 shows the martensite microstructures and statistics of the P1, P2V1, P3 samples. All the samples were mainly composed of parallel lath-shaped martensite with fine spherical or short rod-like eutectic structures between them. With the increase in the energy input, the thickness of the martensite lath decreased (Figure 4g). When the energy input was 85.71 J/mm^3^, the thickness of the martensitic lath ranged from 0.10 to 0.30 µm, and the average width was 0.19 µm. When the energy input was 114.29 J/mm^3^, the thickness of the martensitic lath ranged from 0.11 to 0.20 µm, and the average width was 0.14 µm. When the energy input reached 142.86 J/mm^3^, the thickness of the martensitic lath ranged from 0.07 to 0.18 µm, with an average width of 0.12 µm. It was clear that the martensite laths fabricated via the SLM process were much thinner than their as-cast counterparts, and almost thinner than the Cu_51_Zr_14_ [44] and LaB6/Al [45] refined ones.

Khan [46] pointed out that in Cu-based SMAs, the average martensite width *d_m_* and their parent austenite phase *d_β_* satisfied the following equation:

(2) $$d_{m}\;=\;C\;∛{\;d}_{\beta }$$

Roca et al. [47] verified that the average martensite thickness $d_{\mathrm{plate}}$ and the parent austenite phase d satisfied the following equation:

(3) $$d_{\mathrm{plate}}\;=\;0.036\;d$$

Therefore, the thickness of martensite could be attributed to the grain size of austenite, which was refined by the extremely high cooling rate (2.13 to 6.17 × 10^6^ °C/s) [48] in the SLM process. The refinement rate increased with the rise in the energy input, which was also proportional to the cooling rate. According to the Hall–Patch relationship in Cu-based SMAs [49], with the drastic decrease in grain size, the critical stress of martensite transformation will experience an upward trend, which may have an effect on its mechanical properties.

**Figure 5:**
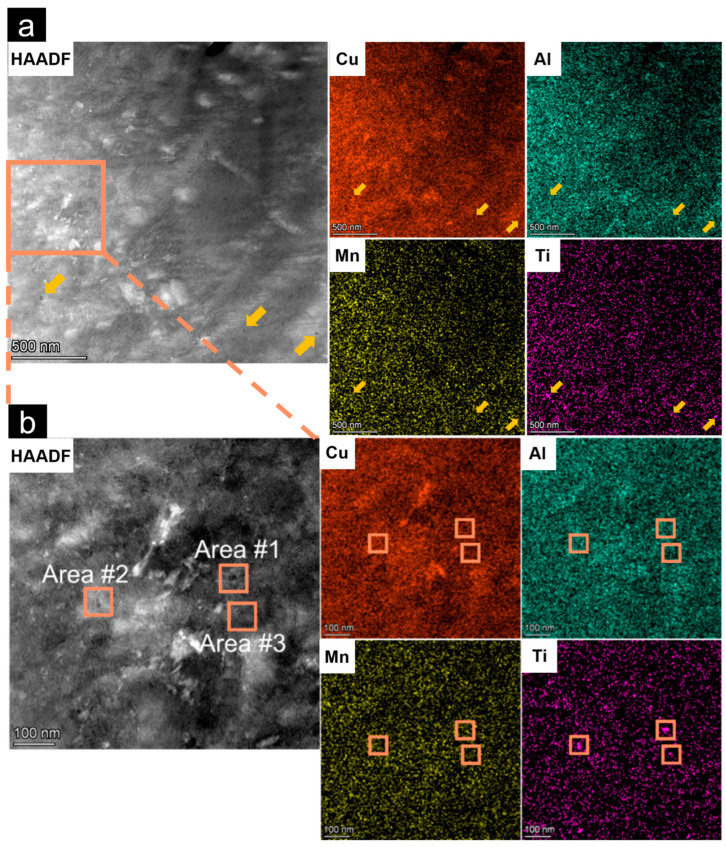
(**a**) STEM images of sample P2V1; (**b**) element distribution of Cu, Al, Mn, and Ti. (Distribution of Cu, Mn, Al, and Ti elements is indicated by red, green, yellow, and purple pixels).

Figure 5 shows the microstructure inspection on the nanoscale with scanning transmission electron microscopy (STEM) and EDS analysis of the P2V1 sample, the yellow arrows and the orange boxes represent the gathering place of elements. Clearly, the dark spherical nanoprecipitates formed homogeneously and densely in the alloy matrix, and there was no obviously aggregated element distribution. The largest diameter of dark particles was 21.64 nm, and the average size of them was approximately 3 to 10 nm. The whole area, as well as dark precipitates area #1, area #2, and area #3, were chosen for the EDS analysis, as listed in Supplementary Table S1. The results revealed that the three precipitates were all enriched in Ti, with a concentration in the range of 0.59 to 1.55 wt.% compared to 0.15 wt.% in the matrix, whereas there was no significant difference in the distribution of the other elements. And the Ti content in the dark nanoparticles (0.59–1.55 wt.%) was significantly higher than in the surrounding matrix (0.15 wt.%), consistent with the stoichiometric presence of Cu_2_TiAl. However, the current evidence linking the dark nanoparticles in Figure 5a to Cu_2_TiAl is indirect.

In order to further analyze the phase structure in the alloy matrix, high-resolution transmission electron microscopy (HR-TEM) was used, as well as selective electron diffraction (SAED) and Fast Fourier transform (FFT).

**Figure 6:**
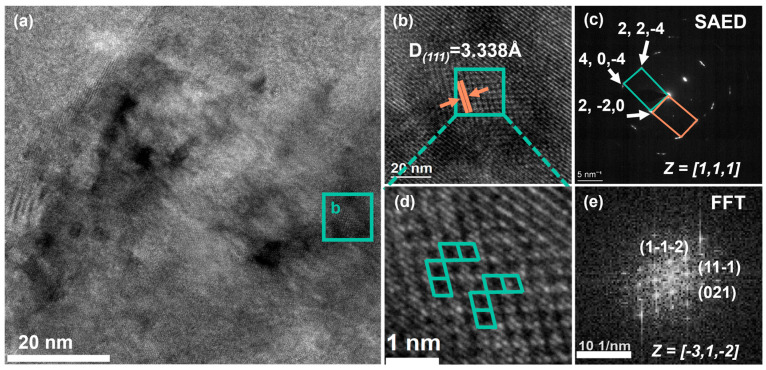
(**a**) HR-TEM image of sample P1V1; (**b**) Partial enlargement of area b in Figure 6a; (**c**) Selected area electron diffraction (SAED) pattern of Figure 6b; (**d**) Partial enlargement of Figure 6b; and (**e**) Fast Fourier Transform (FFT) image of the d area.

High-resolution TEM (HR-TEM) and selected area electron diffraction (SAED) were applied to identify the different phases (Figure 6). High-resolution microscopy shows the significant twinning and number of dislocations in the matrix, as illustrated in Figure 6a,b. The stripe of the twin region in Figure 6b is selected for calculating the lattice spacing, as shown in Figure 6d, determining the crystal surface spacing as D = 3.338 A. The FFT transformation of the region is presented in Figure 6e, with the results further calibrated in Figure 6. According to the analysis results, it can be determined that the twin phase is the Cu_3_Al phase, and that the FFT results show the pattern of [−3, 1, −2] as the axis. The selective electron diffraction pattern shows a complex diffraction pattern in Figure 6c, which was further calibrated to show the Cu_2_AlMn pattern, with the [1, 1, 1] direction as the axis. The reason for this is that Cu_3_Al and Cu_2_AlMn are ordered solid solvents, so the complex diffraction pattern is a multiphase common distribution feature [50].

### 3.3. Phase Identification and Transformation Behavior

**Figure 7:**
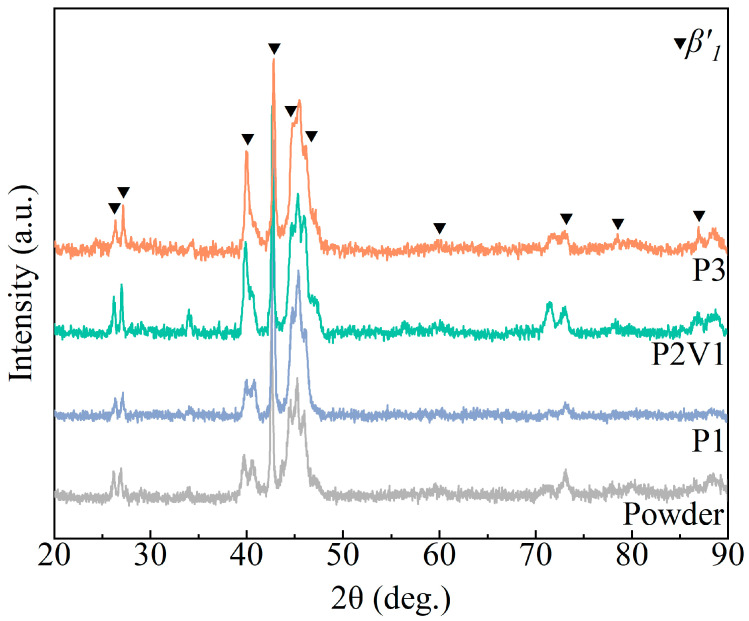
XRD patterns of original powders, P1, P2V1, and P3, as-SLM samples.

Figure 7 displays the X-ray diffraction patterns of the original powders, P1, P2V1, and P3, the as-SLM samples. All the samples indicated a high intensity of $\beta_{1}^{\prime }$ in the martensite phase. It is noteworthy that the (202) peak at a 2θ value of about 40.284° varied from two separated peaks to one high-intensity peak with the increase in the energy input, related to a rise in the degree of crystallinity. No obvious evidence of the *α* and *γ* phases was observed in the XRD patterns of the as-SLM samples, whereas the forming of the *α* phase was common in as-cast Cu-based SMAs, and an aging treatment was necessary to eliminate the unexpected phase [51]. Therefore, the rapid solidification process in SLM has a positive effect on fabricating the single *β* phase.

**Figure 8:**
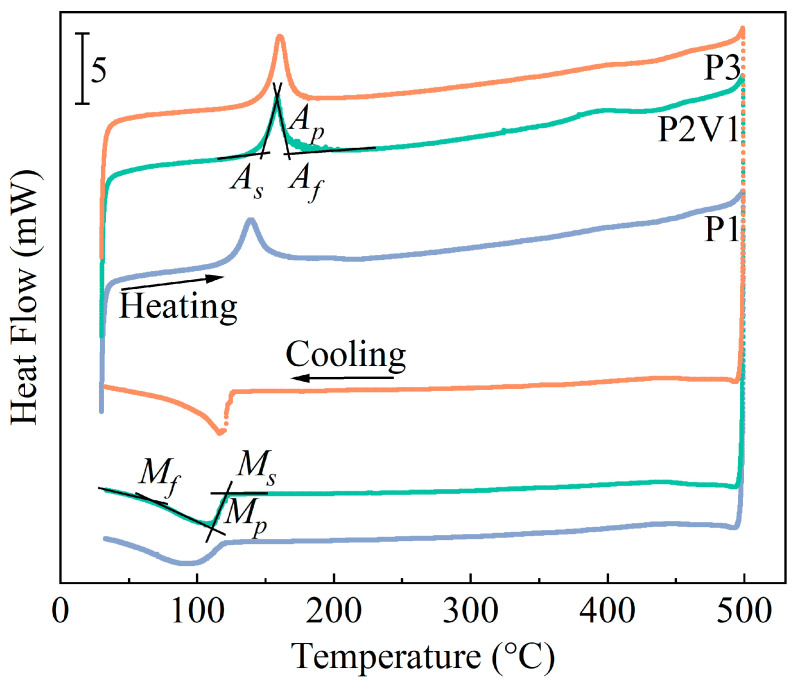
DSC curves of P1, P2V1, and P3 samples.

Figure 8 displays the DSC curves of the P1, P2V1, and P3 samples and the transformation temperature *M_s_*, *M_f_*, *A_s_*, and *A_f_* results, as well as the peak temperature *M_p_*, *A_p_*, and hysteresis temperature *A_f_-M_s_* being listed in Table 3. It can be seen from the DSC curves that only one direct transition and one reverse transition occurred during a heating and cooling cycle. With the rise in the energy input, the *M_s_* decreased, whereas the *A_s_* and hysteresis temperature *A_f_-M_s_* increased. Remarkably, the martensite transformation temperature obtained in this work was much lower than the one calculated from the fitting equation, drawing from the DSC values of the as-cast Cu-Al-Mn SMAs [52]. This is because the martensite transformation temperature declines with the decrease in the martensite lath thickness [47,53], which is consistent with the statistics in Figure 4. In addition, the refinement of martensite lath will lead to an increase in the martensite sub-grain boundaries. This, in turn, elevates the dissipation of energy during transformation [54], resulting in an increase in the hysteresis temperature, and finally reducing the thermal efficiency by prolonging the deformation recovery time. Therefore, optimizing the process parameters during the SLM process is an essential way to adjust the martensitic transformation temperature.

### 3.4. Mechanical Properties

**Figure 9:**
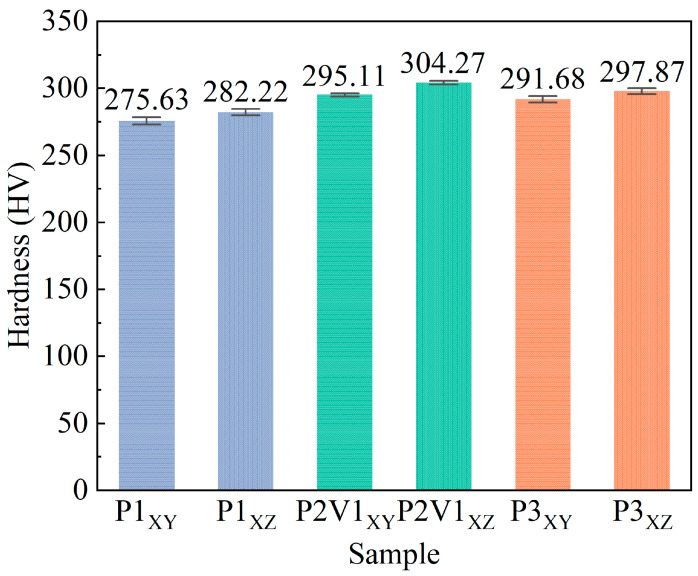
Microhardness of P1, P2V1, and P3 samples in XY and XZ sections.

The microhardness test was taken in different sections of the P1, P2V1, and P3 samples. The XY section, denoting the scanning direction, and the XZ section, representing the building direction, are illustrated in Figure 1. The variation trend of microhardness was in agreement with that of the average density (Figure 9), indicating that the microhardness of the samples was mainly affected by the forming quality. The results showed a significant increase, approximately 40 HV, compared to with the as-cast Cu-11.85Al-2.47Mn SMAs, which consisted not only of the martensite phase, but also the α and γ2 phases [55]. Furthermore, it is important to highlight that a slight difference of approximately 2 to 3% was found in all the samples. This discrepancy indicates that the hardness performance in the XZ section surpassed that of its counterpart, the XY section. As shown in Figure 4 and described in Section 3.2, martensite laths in the XZ direction exhibit a columnar grain morphology, while in the XY direction, they show equiaxed grain features. This directional difference in grain shape is consistent with the thermal gradient characteristics of the SLM process, and leads to mechanical anisotropy. Columnar grain structures typically exhibit higher resistance to plastic deformation than equiaxed grains, due to orientation-dependent dislocation motion barriers, resulting in slightly increased hardness along the build direction [36,47].

The EBSD images demonstrate that the 3D-printed samples exhibit distinct microstructural differences between the building direction (BD) and the scanning direction (SD) due to the directional nature of the selective laser melting process.

**Figure 10:**
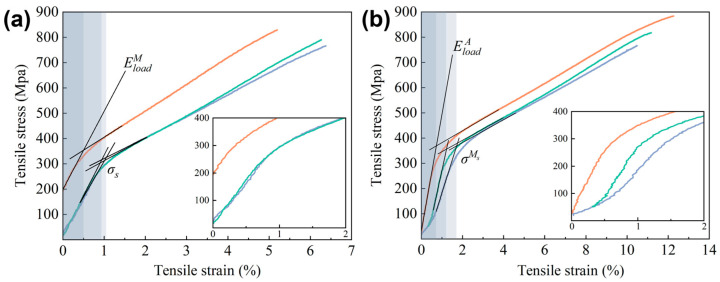
Stress–strain curves of P2V1 samples at (**a**) RT and (**b**) HT.

The uniaxial tensile test was conducted on the sample P2V1, which had the highest average relative density among the samples at room temperature (RT) and 200 °C (HT). This is illustrated in the stress–strain curves in Figure 10a,b. The average ultimate tensile strength ($\sigma_{\mathrm{av}}$) and average elongation at break ($\varepsilon_{\mathrm{av}}$) of the samples were evaluated and are listed in Table 4. According to the DSC curves in Figure 8, the detwinning process of the martensite phase would occur at RT, while stress-induced martensite transformation (SIMT) would occur at HT during the deformation procedure.

**Figure 11:**
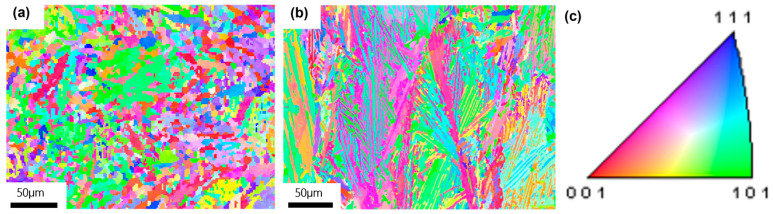
EBSD image of the sample under P2V1 parameter: (**a**) XY plane; (**b**) XZ plane; and (**c**) texture orientation color schematic.

As shown in Figure 11, the elastic deformation occurred at first at RT, and the slope of the curve in this stage could be used to evaluate the elastic modulus of martensite $E_{\mathrm{load}}^{M}\;$ [13]; the average data was 30.38 GPa. After that, the detwinning stage started and the intersection of the slope of the two stages represented the critical stress $\sigma_{s}$ of the martensitic detwinning process, which was 328.32 MPa on average. The samples finally fractured at an average elongation of 5.95% under an average ultimate tensile strength of 795.20 MPa.

However, compared to RT, it is obvious that a yield stage appeared at the beginning of the curves at HT, as illustrated in Figure 10b. This suggests plastic deformation in this period. Then, the elastic deformation started, during which the curve slope could assess the elastic modulus of austenite $E_{\mathrm{load}}^{A}$, valuing 36.85 GPa on average. The following stage witnessed the SIMT process—the intersection of the slope of this and the previous stages referred to the critical stress $\sigma^{M_{s}}$ of the SIMT procedure, which was 365.49 MPa on average. The average elongation at break at HT was 11.30%, almost twice that at RT. In addition, the average ultimate tensile strength was 822.74 MPa, showing a slight increase compared to its counterpart at RT. Two reasons are responsible for the huge increase in elongation at different temperatures. Firstly, a higher elastic modulus was found in austenite, resulting in larger elastic deformation. Meanwhile, SIMT was induced at HT, significantly prolonging and promoting macroscopic inelastic deformation because of the shear strain between atoms. In addition, the enhancement of the ultimate tensile strength at different temperatures could be attributed to the transformation from FCC structural martensite to BCC structural austenite. It is noticeable that in comparison with the as-SLM Cu-13.5Al-4Ni-0.5Ti SMAs with 7.63 ± 0.39% elongation and 541 ± 26 MPa strength at RT, as well as 10.78 ± 1.87% elongation and 611 ± 9 MPa strength at HT24, the as-SLM Cu-11.85Al-3.2Mn-0.1Ti SMAs achieved a higher ultimate tensile strength at both RT and HT, with an increase of more than 200 MPa. This was mostly from Ti-rich nano-precipitation strengthening, which prevented dislocation glide by forming a dense precipitation network in the alloy matrix [56].

**Figure 12:**
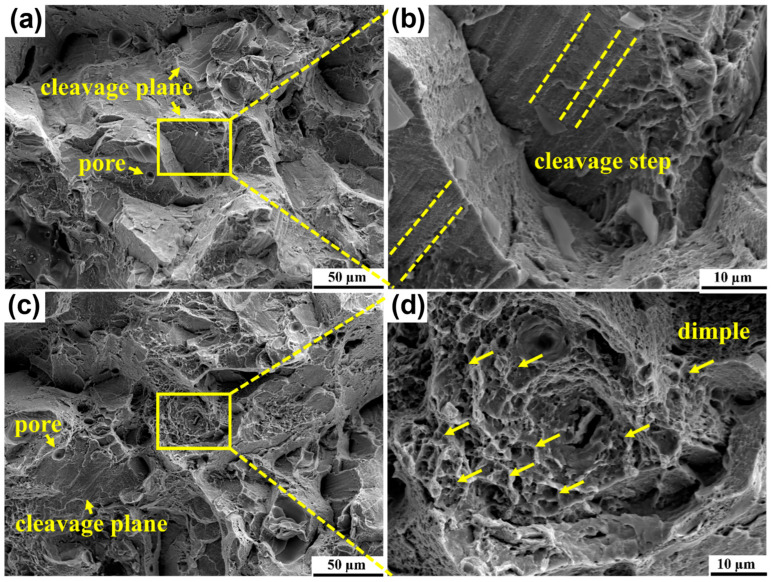
Fracture morphologies of tensile samples: (**a**) fracture at RT; (**b**) morphology of cleavage steps at RT; (**c**) fracture at HT; and (**d**) morphology of dimples steps at HT.

Figure 12 exhibits the fracture morphologies of the tensile samples at RT and HT. Obviously, the brittle fracture characteristics were observed in RT samples with plenty of cleavage planes and cleavage steps, whereas no evidence showed ductile fracture characteristics, as seen in Figure 12a,b. However, apparent ductile fracture characteristics could be found in HT samples, with a great number of dimples on the fracture surface, as illustrated in Figure 12c,d. Apart from that, cleavage planes were also found in the HT samples, indicating that brittle fracture as well as ductile fracture occurred during the deformation process in the SIMT procedure.

## 4. Conclusions

A Cu-11.85Al-3.2Mn-0.1Ti shape memory alloy was successfully fabricated via selective laser melting technology, and the average density could be 99.19% under the optimal parameters (laser power 80 W, scanning speed 200 mm/s, hatch space 0.1 mm, layer thickness 0.035 mm, and energy input 114.29 J/mm^3^). The selective laser melting of martensitic plates results in a distinct “equiaxed crystal” arrangement along the scanning direction, and a “longitudinal arrangement” along the forming direction, leading to a 2–3% higher anisotropic difference in the microhardness in the forming direction compared to that in the scanning direction. The alloy strengthening mechanism resulting from nanoscale precipitation leads to a maximum tensile strength of 829.07 MPa and a fracture strain of 6.38% at room temperature. At 200 °C, it exhibits a maximum tensile strength of 883.68 MPa, with a fracture strain of 12.26%. Compared to the existing SLM additive-manufactured, four-component, copper-based shape memory alloy, this work displays a nearly 30% increment in the maximum tensile strength.

## Figures and Tables

**Table 1:** The nominal chemical composition of the Cu-11.85Al-3.2Mn-0.1Ti original powders (wt.%).

Element	Al	Mn	Ti	Cu
Composition (wt.%)	11.7	3.28	0.17	Ball.

**Table 2:** SLM parameters, volumetric energy input, and average relative density of SLM bulk samples.

Parameters	P (W)	*v* (mm/s)	*h* (mm)	*l* (mm)	*E* (J/mm^3^)	$\rho_{\mathrm{av}}\;$(%)
P1V1	60	200	0.1	0.035	85.71	95.430
P2V1	80	200	0.1	0.035	114.29	99.190
P2V2	80	400	0.065	0.035	87.91	95.197
P2V3	80	600	0.04	0.035	95.24	95.768
P2V4	80	800	0.04	0.035	71.43	92.846
P2V5	80	1000	0.032	0.035	71.43	91.885
P2L2	80	200	0.1	0.025	160	98.220
P2H2	80	200	0.088	0.035	129.87	98.707
P3V1	100	200	0.1	0.035	142.86	98.287
P4V1	120	200	0.1	0.035	171.43	97.974

**Table 3:** Transformation temperature of P1, P2V1, and P3 samples (°C).

No.	M_s_	M_f_	M_p_	A_s_	A_f_	A_p_	A_f_-M_s_
P1	123.26	96.550	97.78	126.54	155.71	139.28	32.45
P2V1	121.20	65.33	110.52	147.50	166.81	158.59	45.61
P3	120.37	55.47	117.91	150.78	169.68	160.23	49.31

**Table 4:** The elastic modulus, critical stress, average ultimate tensile strength, and average elongation at break of P2V1 samples at RT and HT.

**Treatment**	$E_{\mathrm{load}}^{M}/E_{\mathrm{load}}^{A}$ **(GPa)**	$\sigma_{s}/\sigma^{M_{s}}$ (MPa)	$\varepsilon_{\mathrm{av}}$ (%)	$\sigma_{\mathrm{av}}$ (MPa)
RT	30.38 ± 1.85	328.32 ± 23.07	5.95 ± 0.65	795.20 ± 31.66
HT	36.85 ± 10.09	365.49 ± 7.66	11.30 ± 0.90	822.74 ± 58.39

## Data Availability

The original contributions presented in this study are included in the article/Supplementary Material. Further inquiries can be directed to the corresponding author.

## Supplementary Materials

The following supporting information can be downloaded at: https://www.mdpi.com/article/10.3390/mi16080857/s1, Figure S1. (a) Group-A at different processing parameters in the XY plane; (b) the morphologies of the representative samples: weak-melted (80 W,1000 mm/s), well-melted (80 W, 100 mm/s), and excessive-melted (180 W, 400 mm/s). Table S1. EDS analysis result of area #1, area #2, area #3, and the whole area of Figure 5.

## References

[1] Cisse C., Zaki W., Ben Zineb T. (2016). A review of constitutive models and modeling techniques for shape memory alloys. Int. J. Plast..

[2] Alagha A.N., Hussain S., Zaki W. (2021). Additive manufacturing of shape memory alloys: A review with emphasis on powder bed systems. Mater. Des..

[3] Patoor E., Lagoudas D.C., Entchev P.B., Brinson L.C., Gao X.J. (2006). Shape memory alloys, Part I: General properties and modeling of single crystals. Mech. Mater..

[4] Karaca H.E., Karaman I., Basaran B., Lagoudas D.C., Chumlyakov Y.I., Maier H.J. (2006). One-way shape memory effect due to stress-assisted magnetic field-induced phase transformation in Ni2MnGa magnetic shape memory alloys. Scr. Mater..

[5] Costanza G., Tata M.E. (2020). Shape Memory Alloys for Aerospace, Recent Developments, and New Applications: A Short Review. Materials.

[6] Williams E.A., Shaw G., Elahinia M. (2010). Control of an automotive shape memory alloy mirror actuator. Mechatronics.

[7] Wang Y., Venezuela J., Dargusch M. (2021). Biodegradable shape memory alloys: Progress and prospects. Biomaterials.

[8] Ades C.J., Dilibal S., Engeberg E.D. (2020). Shape memory alloy tube actuators inherently enable internal fluidic cooling for a robotic finger under force control. Smart Mater. Struct..

[9] Imran M., Zhang X. (2021). Reduced dimensions elastocaloric materials: A route towards miniaturized refrigeration. Mater. Design.

[10] Qian S., Geng Y., Wang Y., Ling J., Hwang Y., Radermacher R., Takeuchi I., Cui J. (2016). A review of elastocaloric cooling: Materials, cycles and system integrations. Int. J. Refrig..

[11] Hwang D., Lee J., Kim K. (2017). On the design of a miniature haptic ring for cutaneous force feedback using shape memory alloy actuators. Smart Mater. Struct..

[12] Hua S., Liu Y., Jiang J., Xiao F., Hou R., Li A., Cai X., Hu X., Ding X., Jin X. (2023). Shape memory alloy based smart compression stocking and real-time health monitoring app for deep venous thrombosis. Smart Mater. Struct..

[13] Lagoudas D.C. (2008). Shape Memory Alloys.

[14] Babacan N., Pauly S., Gustmann T. (2021). Laser powder bed fusion of a superelastic Cu-Al-Mn shape memory alloy. Mater. Des..

[15] Jiao Z., Wang Q., Yin F., Zhang J., Liu L., Ji P., Chu H., Yu J. (2022). Novel laminated multi-layer graphene/Cu–Al–Mn composites with ultrahigh damping capacity and superior tensile mechanical properties. Carbon.

[16] Wu M.-W., Hu Z.-F., Yang B.-B., Tao Y., Liu R.-P., Ma C.-M., Zhang L. (2023). Additive manufacturing of Cu–Al–Mn shape memory alloy with enhanced superelasticity. Rare Met..

[17] Sutou Y., Omori T., Wang J.J., Kainuma R., Ishida K. (2004). Characteristics of Cu–Al–Mn-based shape memory alloys and their applications. Mater. Sci. Eng. A.

[18] Agrawal A., Dube R.K. (2018). Methods of fabricating Cu-Al-Ni shape memory alloys. J. Alloys Compd..

[19] Gómez-Cortés J.F., Nó M.L., Chuvilin A., Ruiz-Larrea I., San Juan J.M. (2023). Thermal Stability of Cu-Al-Ni Shape Memory Alloy Thin Films Obtained by Nanometer Multilayer Deposition. Nanomaterials.

[20] Zhang Y., Xu L., Zhao L., Lin D., Liu M., Chen W., Han Y. (2023). Deformation mechanism of Cu-Al-Ni shape memory alloys fabricated via laser powder bed fusion: Tension-compression asymmetry. J. Mater. Sci. Technol..

[21] Alaneme K.K., Umar S. (2018). Mechanical behaviour and damping properties of Ni modified Cu–Zn–Al shape memory alloys. J. Sci. Adv. Mater. Devices.

[22] Iacoviello F., Di Cocco V., Natali S., Brotzu A. (2018). Grain size and loading conditions influence on fatigue crack propagation in a Cu-Zn-Al shape memory alloy. Int. J. Fatigue.

[23] Mahdi M.M., Ali A.M., Alalousi M.A., Kadhim D.A., Abid M.A. (2024). Developing a copper-zinc-aluminum alloying technique by vacuum thermal deposition after irradiation by gamma rays (NaI (Ti)) with stabilized zinc metal. Vacuum.

[24] Yuan B., Zhu X., Zhang X., Qian M. (2019). Elastocaloric effect with small hysteresis in bamboo-grained Cu–Al–Mn microwires. J. Mater. Sci..

[25] Yuan B., Qian M., Zhang X., Geng L. (2020). Grain structure related inhomogeneous elastocaloric effects in Cu–Al–Mn shape memory microwires. Scr. Mater..

[26] Yuan B., Zhong S., Qian M., Zhang X., Geng L. (2021). Elastocaloric effect in bamboo-grained Cu71.1Al17.2Mn11.7 microwires. J. Alloys Compd..

[27] Liu J.-L., Huang H.-Y., Xie J.-X. (2015). Superelastic anisotropy characteristics of columnar-grained Cu–Al–Mn shape memory alloys and its potential applications. Mater. Des..

[28] Xu S., Huang H.-Y., Xie J., Takekawa S., Xu X., Omori T., Kainuma R. (2016). Giant elastocaloric effect covering wide temperature range in columnar-grained Cu71.5Al17.5Mn11 shape memory alloy. APL Mater..

[29] Li H., Yuan B., Gao Y., Zhao Y. (2019). Effect of oligocrystallinity on damping and pseudoelasticity of oligocrystalline Cu-Al-Mn shape memory foams. J. Alloys Compd..

[30] Li H., Yuan B., Gao Y. (2018). Achieving high oligocrystalline degree via strut architecture tailoring to increase the damping and mechanical properties of spherical porous CuAlMn SMAs. J. Alloys Compd..

[31] Zheng S., Chen S., Hong S., Su Z., Zhang J., Wang L., Wang C., Yang S. (2022). Microstructure, martensitic transformation and excellent shape memory effect of Ga-alloyed Cu–Al–Mn–Fe shape-memory single crystal. J. Sci. Adv. Mater. Devices.

[32] Xin C., Zhao X., Geng H., Hao L., Li Y., Chen T., Gong P. (2023). Microstructure, grain and nanowire growth during selective laser melting of Ag–Cu/diamond composites. RSC Adv..

[33] Chen Q., Jing Y., Yin J., Li Z., Xiong W., Gong P., Zhang L., Li S., Pan R., Zhao X. (2023). High Reflectivity and Thermal Conductivity Ag–Cu Multi-Material Structures Fabricated via Laser Powder Bed Fusion: Formation Mechanisms, Interfacial Characteristics, and Molten Pool Behavior. Micromachines.

[34] Gustmann T., Neves A., Kühn U., Gargarella P., Kiminami C.S., Bolfarini C., Eckert J., Pauly S. (2016). Influence of processing parameters on the fabrication of a Cu-Al-Ni-Mn shape-memory alloy by selective laser melting. Addit. Manuf..

[35] Gustmann T., Schwab H., Kühn U., Pauly S. (2018). Selective laser remelting of an additively manufactured Cu–Al–Ni–Mn shape-memory alloy. Mater. Des..

[36] Tian J., Zhu W., Wei Q., Wen S., Li S., Song B., Shi Y. (2019). Process optimization, microstructures and mechanical properties of a Cu-based shape memory alloy fabricated by selective laser melting. J. Alloys Compd..

[37] Zhuo L., Song B., Li R., Wei Q., Yan C., Shi Y. (2020). Effect of element evaporation on the microstructure and properties of CuZnAl shape memory alloys prepared by selective laser melting. Opt. Laser Technol..

[38] Abolhasani D., Han S.W., VanTyne C.J., Kang N., Moon Y.H. (2021). Enhancing the shape memory effect of Cu–Al–Ni alloys via partial reinforcement by alumina through selective laser melting. J. Mater. Res. Technol..

[39] Abolhasani D., Moon B., Kang N., VanTyne C.J., Moon Y.H. (2023). High-performance Cu-Al shape memory alloy in ternary combination with graphene fabricated by powder bed fusion process. J. Alloys Compd..

[40] Abolhasani D., Han S.W., VanTyne C.J., Kang N., Moon Y.H. (2022). Powder bed fusion of two-functional Cu–Al–Ni shape memory alloys utilized for 4D printing. J. Alloys Compd..

[41] Dang M., Xiang H., Li J., Cai C., Wei Q. (2023). Laser powder bed fusion of full martensite Cu–Al–Mn–Ti alloy with good superelasticity and shape memory effect. Mater. Sci. Eng. A.

[42] Yin J., Zhang W., Ke L., Wei H., Wang D., Yang L., Zhu H., Dong P., Wang G., Zeng X. (2021). Vaporization of alloying elements and explosion behavior during laser powder bed fusion of Cu–10Zn alloy. Int. J. Mach. Tools Manuf..

[43] King W.E., Barth H.D., Castillo V.M., Gallegos G.F., Gibbs J.W., Hahn D.E., Kamath C., Rubenchik A.M. (2014). Observation of keyhole-mode laser melting in laser powder-bed fusion additive manufacturing. J. Mater. Process. Technol..

[44] Yang J., Wang Q.Z., Yin F.X., Cui C.X., Ji P.G., Li B. (2016). Effects of grain refinement on the structure and properties of a CuAlMn shape memory alloy. Mater. Sci. Eng. A.

[45] Liu X., Wang Q., Kondrat’ev S.Y., Ji P., Yin F., Cui C., Hao G. (2019). Microstructural, Mechanical, and Damping Properties of a Cu-Based Shape Memory Alloy Refined by an In Situ LaB6/Al Inoculant. Metall. Mater. Trans. A.

[46] Khan A.Q. (1974). The application and interpretation of the “time law” to the growth of β grain size and martensite plate thickness in copper-based martensites. J. Mater. Sci..

[47] La Roca P., Isola L., Vermaut P., Malarría J. (2015). Relationship between martensitic plate size and austenitic grain size in martensitic transformations. Appl. Phys. Lett..

[48] Li Y., Gu D. (2014). Parametric analysis of thermal behavior during selective laser melting additive manufacturing of aluminum alloy powder. Mater. Des..

[49] Khan A.Q., Brabers M., Delaey L. (1974). The Hall-Petch relationship in copper-based martensites. Mater. Sci. Eng..

[50] Alés A., Lanzini F. (2020). Mechanical and thermodynamical properties of β–Cu–Al–Mn alloys along the Cu3Al → Cu2AlMn compositional line. Solid State Commun..

[51] Yong P., Zhu X., Yanlin J., Rui Z., Jiang Y., Wenting Q., Zhou L. (2020). Hot deformation behavior of a CuAlMn shape memory alloy. J. Alloys Compd..

[52] Wang H., Huang H.-Y., Su Y.-J. (2020). Tuning the operation temperature window of the elastocaloric effect in Cu–Al–Mn shape memory alloys by composition design. J. Alloys Compd..

[53] Sutou Y., Omori T., Yamauchi K., Ono N., Kainuma R., Ishida K. (2005). Effect of grain size and texture on pseudoelasticity in Cu–Al–Mn-based shape memory wire. Acta. Mater..

[54] Roca P.L., Isola L., Vermaut P., Malarría J. (2017). Relationship between grain size and thermal hysteresis of martensitic transformations in Cu-based shape memory alloys. Scr. Mater..

[55] Jiao Z., Wang Q., Yin F., Cui C. (2018). Effect of precipitation during parent phase aging on the microstructure and properties of a refined CuAlMn shape memory alloy. Mater. Sci. Eng. A.

[56] Pan R.C., Fan D., Bian Y.L., Zhao X.J., Zhang N.B., Lu L., Cai Y., Luo S.N. (2023). Effect of minor elements Al and Ti on dynamic deformation and fracture of CoCrNi-based medium-entropy alloys. Mater. Sci. Eng. A.

