# Supplementary material for: Fabrication of Cu-Al-Mn-Ti Shape Memory Alloys via Selective Laser Melting and Its Nano-Precipitation Strengthening
Source: Micromachines (Basel). 2025 Jul 25;16(8):857. doi: 10.3390/mi16080857 (PMC12388566; doi:10.3390/mi16080857)
Supplement: Supplementary file 1 [file micromachines-16-00857-s001.zip › micromachines-3705728-supplementary.pdf]

Fabrication of Cu-Al-Mn-Ti Shape Memory Alloys via Selective Laser Melting and Its Nano-Precipitation Strengthening

Lijun He [1], Yan Li [2,3,*], Qing Su [3], Xiya Zhao [3] and Zhenyu Jiang [3]

[1] Hubei Engineering Research Center for BDS-Cloud High-Precision Deformation Monitoring, Artificial Intelligence School, Wuchang University of Technology, Wuhan 430223, China
[2] Institute for Advanced Marine Research, China University of Geosciences, Guangzhou 511462, China
[3] Gemmological Institute, China University of Geosciences, Wuhan 430074, China
* Correspondence: yanli@cug.edu.cn

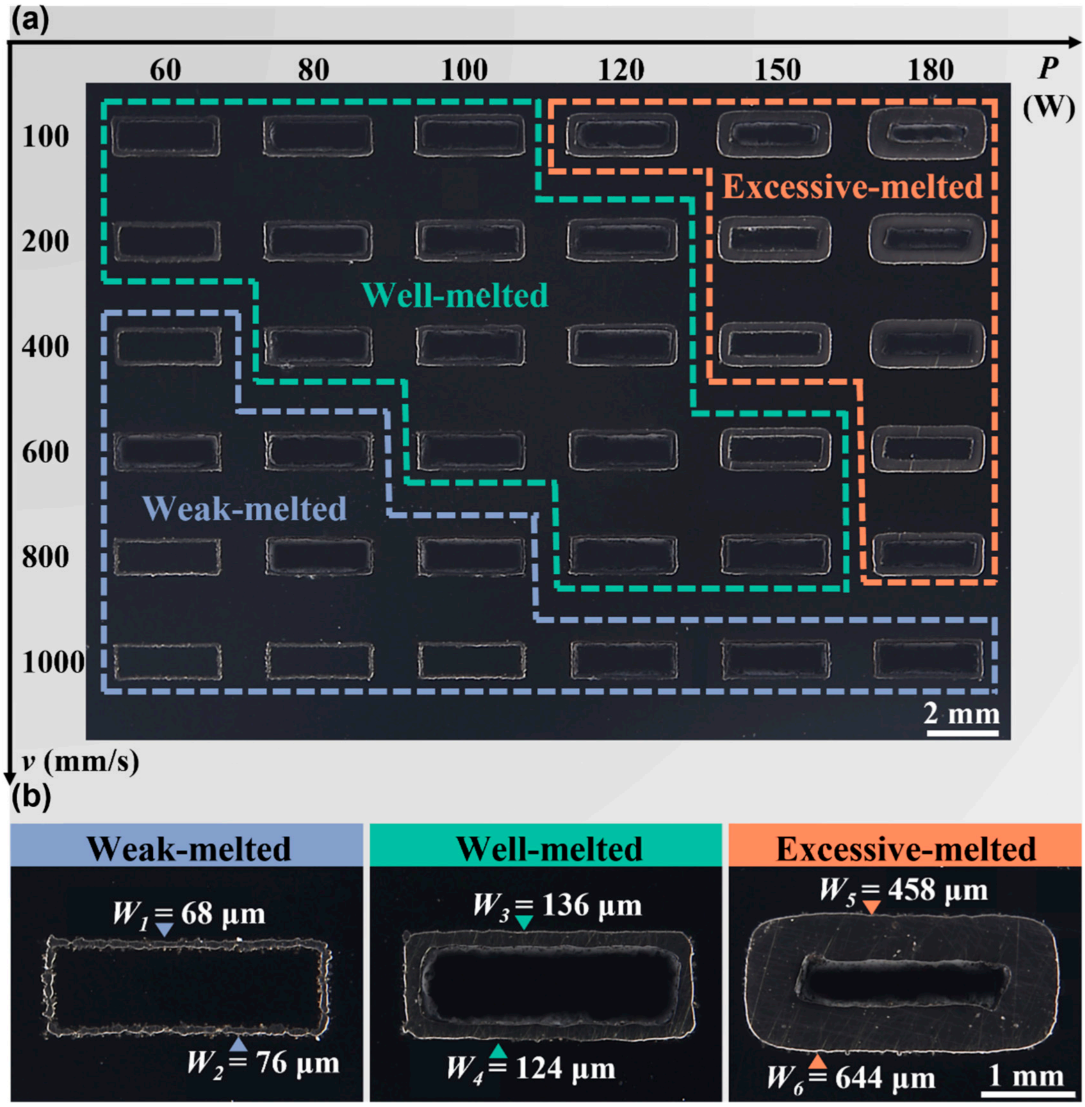

Figure S1. (a) Group-A at different processing parameters in the XY plane; (b) the morphologies of the representative samples: weak-melted (80 W,1000 mm/s), well-melted (80 W, 100 mm/s), and excessive-melted (180 W, 400 mm/s).

Table S1. EDS analysis result of area#1, area#2, area#3 and the whole area of Fig.5.

	Element	Family	Automic Fraction (%)	Automic Error (%)	Mass Fraction (%)	Mass Error (%)	Fit Error (%)
Area #1	Ti	K	1.81	0.50	1.55	0.35	14.45
	Mn	K	2.90	0.73	2.84	0.57	9.23
	Al	K	18.84	3.70	9.06	1.10	3.64
	Cu	K	76.44	17.93	86.56	15.28	0.69

Area #2	Ti	K	1.44	0.40	1.22	0.28	15.14
	Mn	K	2.50	0.64	2.43	0.49	9.74
	Al	K	17.87	3.62	8.52	1.09	4.97
	Cu	K	78.19	18.56	87.83	15.60	0.67
Area #3	Ti	K	0.68	0.30	0.59	0.24	36.56
	Mn	K	2.51	0.65	2.48	0.52	11.12
	Al	K	20.39	3.99	9.86	1.18	2.70
	Cu	K	76.42	17.98	87.08	15.42	0.76
Total	Ti	K	0.18	0.04	0.15	0.03	0.98
	Mn	K	2.48	0.59	2.40	0.43	0.13
	Al	K	18.07	3.58	8.60	1.02	0.03
	Cu	K	79.26	18.92	88.84	15.84	0.04
